# Socio-Economic Differentials in Impoverishment Effects of Out-of-Pocket Health Expenditure in China and India: Evidence from WHO SAGE

**DOI:** 10.1371/journal.pone.0135051

**Published:** 2015-08-13

**Authors:** Kaushalendra Kumar, Ashish Singh, Santosh Kumar, Faujdar Ram, Abhishek Singh, Usha Ram, Joel Negin, Paul R. Kowal

**Affiliations:** 1 International Institute for Population Sciences, Mumbai, India; 2 Indian Institute of Technology Bombay, Mumbai, India; 3 Sam Houston State University, Houston, United States of America; 4 School of Public Health, University of Sydney, Sydney, Australia; 5 World Health Organization (WHO), Geneva, Switzerland; University of Washington, UNITED STATES

## Abstract

**Background and Objectives:**

The provision of affordable health care is generally considered a fundamental goal of a welfare state. In addition to its role in maintaining and improving the health status of individuals and households, it impacts the economic prosperity of a society through its positive effects on labor productivity. Given this context, this paper assesses socioeconomic-differentials in the impact of out-of-pocket-health-expenditure (OOPHE) on impoverishment in China and India, two of the fastest growing economies of the world.

**Data and Methods:**

The paper uses data from the World Health Organisation’s Study on Global Ageing and Adult Health (WHO SAGE), and Bivariate as well as Multivariate analyses for investigating the socioeconomic-differentials in the impact of out-of-pocket-health-expenditure (OOPHE) on impoverishment in China and India.

**Results and Conclusions:**

Annually, about 7% and 8% of the population in China and India, respectively, fall in poverty due to OOPHE. Also, the percentage shortfall in income for the population from poverty line due to OOPHE is 2% in China and 1.3% in India. Further, findings from the multivariate analysis indicate that lower wealth status and inpatient as well as outpatient care increase the odds of falling below poverty line significantly (with the extent much higher in the case of in-patient care) due to OOPHE in both China and India. In addition, having at least an under-5 child in the household, living in rural areas and having a household head with no formal education increases the odds of falling below poverty line significantly (compared to a head with college level education) due to OOPHE in China; whereas having at least an under-5 child, not having health insurance and residing in rural areas increases the odds of becoming poor significantly due to OOPHE in India.

## Introduction

The provision of affordable health care is generally considered a fundamental goal of a welfare state. In addition to its role in maintaining and improving the health status of individuals and households, it impacts the economic prosperity of a society through its positive effects on labor productivity. The affordability of a health care system is often conceptualized in terms of “financial protection”, that is, individuals and households should be protected from incurring a burden of health care expenditure that would adversely affect their economic wellbeing [1].

Nevertheless, policies in many countries compel households and individuals to cover a substantial portion of healthcare costs out-of-pocket. The annual mean per capita health expenditure was Int$1080 (purchasing power parity [PPP] estimate) globally in 2011 and varied from Int$68 in low income to Int$647 in middle income countries. Of the total health expenditure, per capita government contribution in 2011 was Int$623 worldwide, Int$27 in low income countries and Int$361 in middle income countries, respectively, whereas, out-of-pocket health expenditure (OOPHE) constituted 21% of the total health expenditure in 2011 globally. The percentage of health expenditure covered by OOPHE varied from 47% in low income countries to 34% in middle income countries [2].

In countries where a major part of health care is financed by OOPHE, health expenditures can have impoverishing effects on the economic status of households, especially among the poorer socioeconomic strata [1,3–8]. For example, households in the lowest income quintile and/or with higher inpatient expenses are more likely to borrow or sell assets in order to cope with health care expenses [9]. One analysis of health care financing strategy in 40 low and middle income countries by Kruk, Goldmann, and Galea (2009) revealed that about 26% (one billion) of households borrow or sell their assets to pay for health care [10]. From a policy perspective, health care financing in the absence of any other health security inflates the household consumption expenditure and hence underestimates the actual level of poverty in countries [11].

Though the research on catastrophic health expenditures (CHE) is fairly developed and there are a few studies examining the impoverishment effects of OOPHE across different countries [12–18], detailed analyses of socioeconomic differentials in the impoverishment effects of OOPHE are rare. Therefore, in this paper, we fill this gap by investigating socioeconomic differentials in the impact of OOPHE on impoverishment in China and India. Li et al. (2014) and Li et al. (2012) investigate the extent of CHE and impoverishment from medical expenses in China, but their main focus is on the determinants of CHE rather than the determinants of impoverishment effects [15,16]. Similarly, studies by Balarajan et al. (2011) and Garg and Karan (2009) are limited to the estimation of overall poverty level increase due to OOPHE in India [12,13]. In contrast, we use data from two heavily populated countries China and India to provide evidence on socioeconomic differentials in the share of OOPHE in total and non-food expenditures, the percentage of the population falling below poverty line (poverty head count) due to OOHPE, the average poverty gap due to OOPHE, and the odds of becoming poor due to OOPHE.

Our choice of China and India is motivated by several considerations. First, China and India are not only the two most populous countries in the world but are also among the fastest growing economies of the present times. Second, among the developing countries, financial hardship of health payments is reportedly higher in China and India, with households relying excessively on OOPHE [17]. Finally, a substantial proportion of the population falls below the poverty line due to OOPHE in these countries. For example, taking a poverty line of US$1.08, Van Doorslaer et al., (2006) estimated that about 2.6% (32 million) and 3.7% (37–39 million) of the population in China and India, respectively, fell below the poverty line due to OOPHE in 1999–2000 alone [19]. Similarly, Garg and Karan (2009) estimated that the overall poverty level increased by 3.2% due to OOPHE in India in 1999–2000 and a study by Balarajan et al. (2011) indicated that about 39 million Indians are pushed into poverty by OOPHE every year [12,13]. Regarding China, estimates show that about 7.5% of households became poor due to OOPHE in China in 2008 [14,16].

**Fig 1:**
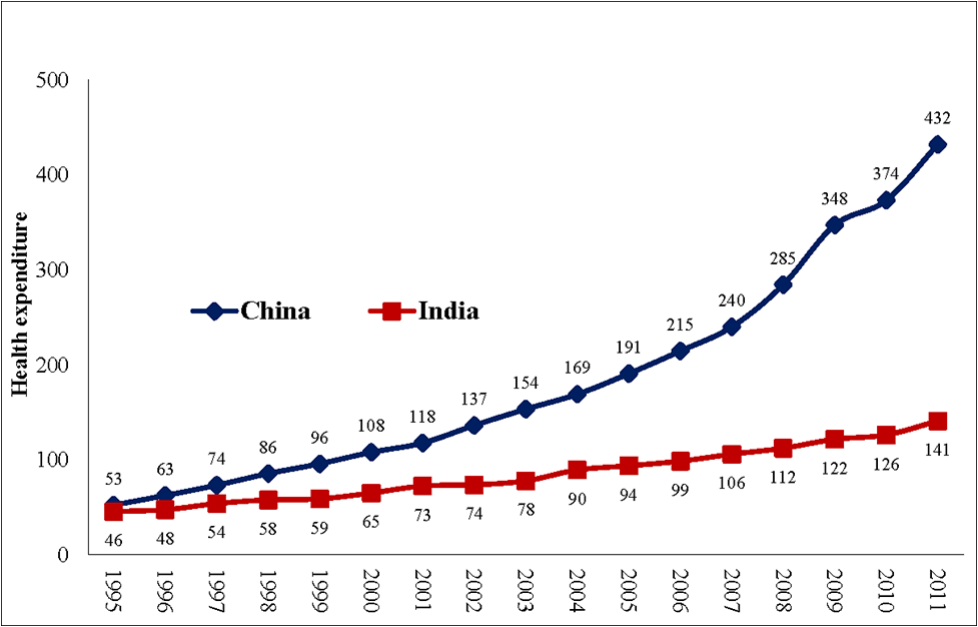
Per capita annual expenditure on health (PPP international $), 1995–2011 (Based on data from WHO 2014).

**Fig 2:**
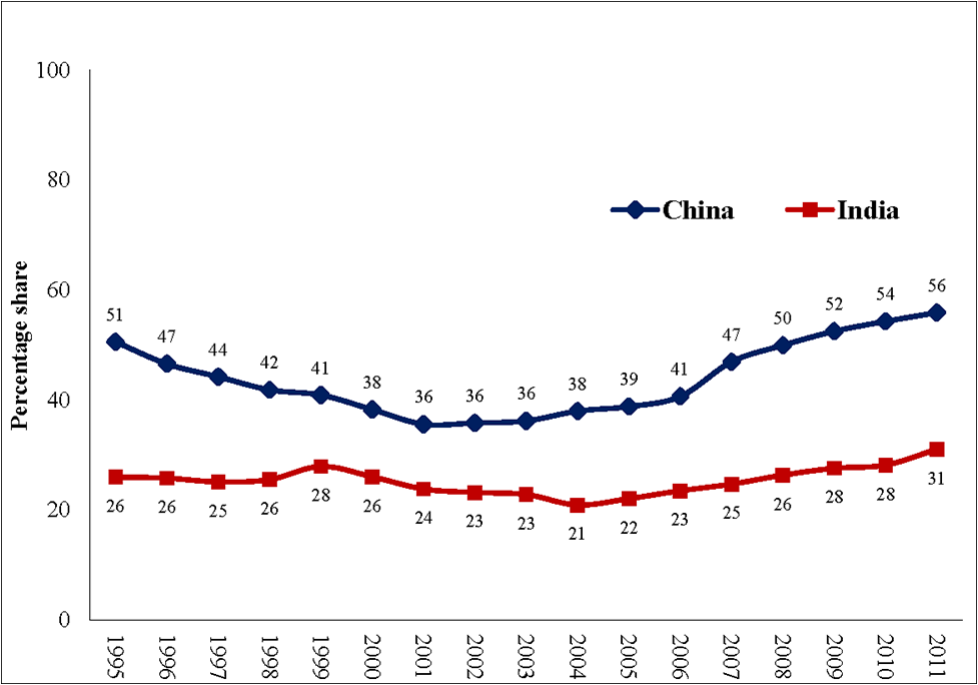
Government expenditure as a percentage of total expenditure on health, 1995–2011 (Based on data from WHO 2014).

**Fig 3:**
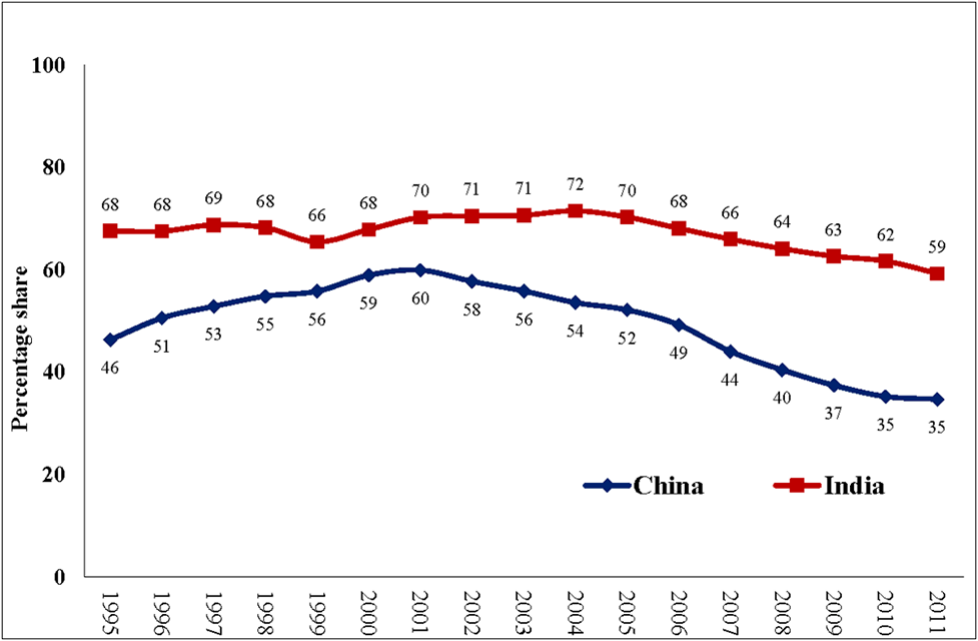
Out-of-pocket health expenditure as a percentage of total expenditure on health, 1995–2011 (Based on data from WHO 2014).

Despite the potentially impoverishing effects of OOPHE, the annual per capita expenditure on health has increased from Int$ 53 (PPP) in 1995 to Int$ 432 (PPP) 2011 in China and from Int$ 46 (PPP) in 1995 to Int$ 141 (PPP) in 2011 in India (Fig 1). This is combined with near stagnation in the share of government expenditure as a percentage of total expenditure on health from 1995 to 2011 in both the countries, the government share being only 31% and 56% in India and China, respectively in 2011 (Fig 2). Nevertheless, the two countries have seen some improvement (China– 46% to 35% and India– 68% to 59%) in the share of OOPHE as a percentage of total health expenditure during the time period from 1995–2011 (Fig 3).

The remainder of the paper is organized as follows: the next two sections briefly describe the data and the methods used in the paper, these are followed by a section summarizing the findings, and the final section provides our main conclusions along with a discussion of the main results.

## Data and Methods

### Ethics statement

The data were analyzed anonymously, using publicly available secondary data; therefore no ethics review is required for this work.

### Data

We use data from the World Health Organization’s Study on Global Ageing and Adult Health (SAGE), which was implemented in China, Ghana, India, Mexico, The Russian Federation and South Africa in the years 2007–2010. The target population in each country was adults (18 years and older), and data were also collected the households of study participants. The survey was primarily aimed to provide nationally comparable estimates on health status, wellbeing and health care utilization by the adult population in each country [20]. The data were collected using face-to-face paper and pencil interviews (PAPI) for India. In China, 50% PAPI and 50% face-to-face computer assisted personal interviews (CAPI) were used [20]. The household (individual) response rates were about 99% (68%) in India to 95% (99%) in China. For further details on sampling and other procedures, refer to Kowal et al. (2012) [20].

While the focus of WHO-SAGE was on older adults, the design of the study allows for robust analysis of health across adulthood (i.e. a life-course perspective to the ageing process). This includes the variables in this paper—where age of household head is used to identify adults (<50) and older adults (50+) for comparison. Comparisons were made at this level, along with a count of older adults and children in the household—two "vulnerable" groups with supporting literature about health care utilization and cost patterns that could contribute a significant amount of the variation in household health expenditure. Expenditure variables are captured at the household level, with assumptions made about how resources are used in households. Individual level analyses require adjustments of expenditure based on household size to configure individual level consumption, and so for these analyses, we believed the variables chosen to assess "age" were the most robust approach to predicting consumption/consumption outcomes. Also, the data is publicly available at: http://apps.who.int/healthinfo/systems/surveydata/index.php/catalog/sage/about

As mentioned above, though the information regarding the impoverishment effects of health payments in the survey is collected at the household level, the analysis has been adjusted using household size (by using appropriate population weight a multiple of sampling weights with household size) to generate population level estimates. In total, SAGE interviewed 10,218 households in China and 9626 households in India. Because our study compares out-of-pocket health expenditure to both total expenditure and non-food expenditure, households for which food expenditure was not reported were excluded from the analysis. The final sample includes 9591 (93.9% of total) households from China and 9583 (99.6% of total) households from India. Wealth quintile wise distribution of the excluded household in China shows that about 36% households belong to the poorest or poor wealth quintile and about 40% households from the richer or richest wealth quintile. Therefore, the above mentioned exclusion of households from the sample is not likely to affect the estimates significantly.

The survey captures household food expenditure for the last 7 days preceding the survey, non-food and health care and services expenditure for the 30 days preceding the survey, and expenditure on health aids, overnight stays and long term health care for the 12 months preceding the survey. To obtain comparable figures, we converted each expenditure measure to a 30-day basis.

### Methods

In this paper we focus mainly on socioeconomic differentials in the share of OOPHE as a proportion both of total and of non-food expenditures (in terms of capacity to pay which has been defined subsequently), the impoverishment effects of OOPHE, and the odds of impoverishment due to OOPHE. It is worth noting that we have followed the definition and concepts of the impoverishment effect as described by [18,21].

Out-of-pocket health expenditure (OOPHE) and its share in total and non-food expenditure (capacity to pay)

Out-of-pocket health expenditure includes the net total of insurance reimbursement (OOPHE includes health insurance premium, therefore we have excluded the health insurance reimbursement from OOPHE) and household expenditure in last 30 days on doctors’ registration and consultation, traditional and alternative healers, diagnostic and laboratory tests, medication, dental care, ambulatory care, and other health care products or services. It also includes the net total of insurance reimbursement and household expenditure in last year on mandatory or voluntary health insurance premiums, health related aids, overnight stay in health facilities, and long-term care.

It is worth noting that the estimate of OOPHE and its impoverishment effects depend on the reference period as well as the number of items (related to health expenditure) on which information has been collected in the survey [22,23]. Comparing different surveys in India Raban et al. [23] shows that the percentage of households incurring CHE is twice as per SAGE 2007–08 survey (31.9%) compared with National Sample Survey’s (NSS) consumption expenditure survey, 2009–10 (13.9%). However, as per WHO SAGE 2007–08 report (using the same threshold), about 23.9% household incur CHE in India [24]. On one hand, it can be taken as WHO SAGE underestimate non-health expenditures relative to more “economics” oriented surveys (like NSS consumption expenditure survey) and thus measure higher rates of catastrophic health expenditure, but on the other hand, the more pertinent question is which estimates should be taken as more reliable. In this regard, Raban et al. [23] itself reports that there are 28 questions on inpatient and outpatient health expenditure in NSS health expenditure survey 2004–05 compared with only 13 questions in the NSS consumer expenditure survey 2004–05. This reflects in 29.2% CHE (almost twice) as per NSS health expenditure survey 2004–05 compared with only 14% CHE in the NSS consumer expenditure survey-2004-05 [23]. Similarly, mean OOP inpatient care payment is US$ 36.8 in the NSS health expenditure survey 2004–05 compared with only US$ 18.7 in the NSS consumer expenditure survey-2004-05 in India [23]. Hence, it can also be said that WHO SAGE or NSS health expenditure surveys are designed to capture health expenditures in a better way compared to other “economics” oriented surveys like NSS consumer expenditure surveys whose focus is more on overall consumption expenditure. [13] can also be seen in this regard.

Unlike many previous studies, ours study includes ambulance charges in the health expenditure figure, which constitutes a large share of total health expenditure in developing countries [1]. Haffner, Moschel, and ten Horn (1987) found that retrospective reports of ambulatory care up to 6 months prior to the survey and outpatient care up to 3 months prior to the survey provide reliable estimates [25].

Capacity to pay (*ctp*) is defined as the household’s expenditure (*exp*) in excess of the subsistence expenditure (*se*),which is the minimum expenditure required to remain on or above the poverty line [18,21].

$$Household\prime s\;capacity\;to\;pay\;\left(ctp_{i}\right)\;=exp_{i}-\;se_{i};\;if\;se_{i}\;\leq \;food_{i}$$

For the household reporting food expenditure less than subsistence expenditure (*food*) then capacity to pay is:

$$Household\prime s\;capacity\;to\;pay\;\left(ctp_{i}\right)\;=\;exp_{i}-\;food_{i};\;if\;se_{i}>food_{i}$$

Subsistence expenditure is the equivalent household size adjusted average food expenditure of the household lying between its 45^th^ and 55^th^ percentile [26]. Household level subsistence expenditure is the poverty line multiplied by equivalent household size.

$$Subsistence\;expenditure\;\left(se_{i}\right)\;=\;pl\;*\;eqsize_{i}$$

Where *pl* and *eqsize*_*i*_ = *hhsize* ^0.56^ are poverty line and equivalent household size of the *i* ^*th*^ household.

The poverty line is the weighted average of the equivalent food expenditures (*eqfood*) in the range of food expenditure (*food*) shares of total household expenditure (*exp*) that are at the 45^th^ (*foodexp*_45_) and 55^th^ (*foodexp*_55_) percentile across the whole sample [21].

$$Poverty\;line\left(pl\right)=\frac{\sum w_{i}\;*\;eqfood_{i}}{\sum w_{i}}$$

Where (*foodexp*_45_)≤ (*eqfood*_*i*_ = *food*_*i*_/*eqsize*_*i*_) ≤ (*foodexp*_55_) and *foodexp*_*i*_ = *food*_*i*_/*exp*_*i*_ is the food expenditure share to the total household expenditure of the *i* ^th^ household.

A household is classified as poor if its total expenditure is less than the subsistence expenditure [21].

#### Impoverishment effects of OOPHE

We use two indices to capture the impoverishment effects of OOPHE: poverty head count ratio and poverty gap ratio. Poverty head count captures households whose net total household expenditure is less than the required subsistence expenditure solely due to OOPHE [21].

$$Poverty\;head\;count\;\left(Impoor_{i}\right)\;=\;1\;if\;exp_{i}\geq \;se_{i}\;and\;\left(exp_{i}-\;OOPHE_{i}\right)<se_{i}$$

‘0’ otherwise; where, *exp*_*i*_ is the total expenditure of the *i* ^*th*^ household.

The poverty headcount ratio is measured as the households who fall below the poverty line (at constant 2007–10 international $ 89.43 in India and international $ 125.67 in China) due to OOPHE as a proportion of all the households in the population [3,19,27].

$$Poverty\;head\;count\;ratio\;\left(HCR\right)=\frac{\sum_{i=1}^{N}Impoor_{i}}{N}$$

The poverty gap measures the percentage deficit from the poverty line of those households that have become poor due to OOPHE, and poverty gap ratio measures the percentage deficit from the poverty line of households that have become poor due to OOPHE as a proportion of all the households in the population. In a sense, the poverty gap ratio measures the average percentage deficit from the poverty line due to OOPHE for the population [3,19,27]

The poverty gap for the *i* ^*th*^ household can be defined as:

$$\begin{array}{l} Poverty\;gap_{i}=Impoor_{i}\;*\;\left\{se_{i}-\left(exp_{i}-OOPHE_{i}\right)\right\}/se_{i}\\ Povery\;gap\;ratio\;\left(PGR\right)=\frac{1}{N}\sum_{i=1}^{N}Poverty\;gap_{i} \end{array}$$

#### Statistical modeling

We modeled associations between selected socioeconomic characteristics and the odds of becoming poor due to OOPHE using logistic regressions. The binary outcome variable was whether or not a household has become poor (net total household expenditure becoming less than the required subsistence expenditure) due to OOPHE.

#### Covariates or independent variables

The catastrophic health expenditure and impoverishment effect of OOPHE depends on demographic and socio-economic condition of the household. Existing studies on the subject have established that household demographic status like age and sex of the household head, number of 50+ aged elderly and 0–5 years aged children, household size and place of residence determine the CHE and impoverishment effect of OOPHE [14,16,28–30]. For a given value of health expenditure, the catastrophic impact of OOPHE depends on the food expenditure which in turn depends on household head’s education and economic status [14,26]. The study Wagstaff and van Doorslaer [3] also find that high impoverishment intensity of OOPHE is due to poor becoming poorer rather than the non-poor falling below poverty line. Further, [31] shows that lower income groups pay a higher share of their income compared to the higher income groups on health care.

In the present study, we have measured the socio-economic condition of households by household head’s education and wealth quintile to which a household belongs. Health insurance coverage reduces the chance of catastrophic of impoverishment effect of OOPHE. Studies also find that household with family member enrolled in any insurance scheme has lower rates of CHE and impoverishment [14,18,30,32]. Inpatient hospitalization of the household member increases the chance of CHE [14]. And Wagstaff and van Doorslaer [3] find that non-hospital expenditure during inpatient care of the household member increases the chance of impoverishment [3]. In the present study, hospitalization is measured in terms of inpatient and outpatient care received by the household members.

Since OOPHE share, poverty head count and poverty gap ratios are continuous outcome, therefore significant statistical socioeconomic difference in impoverishment effect of OOPHE is first tested by performing one-way ANOVA test. The one-way analysis of variance (ANOVA) is used to determine whether there are any significant differences between the means of three or more independent groups. That is, the one-way ANOVA compares the means between the groups we are interested in and determines whether any of those means are significantly different from each other. In order to check does socio-economic differential in impoverishment effect of OOPHE we first run the multivariate logistic regression adjusting for socio-economic status and health insurance of the household member in Model-1. We further adjusted for the household demographic characteristics in Model-2, which gives the demographic characteristics controlled socio-economic differential in impoverishment effect of OOPHE. Finally, Model-3 test show socio-economic status mediates between OOPHE, due to health care utilization, and its impoverishment effect we take interaction of wealth quintile and inpatient or outpatient care received. All bivariate and multivariate analysis is performed using corresponding national weight and taking into account the survey design of the SAGE survey data.

## Results

### Distribution of Socio-economic Characteristics

Table 1 shows the percentage distribution of the population by socioeconomic characteristics. Regarding the educational status of households, India has a higher proportion of population living in households in which the head has no formal schooling (32%, CI: 29.6–34.2)), whereas the corresponding figure in China is only 15% (CI: 13.2–16.6). In terms of health insurance, there is a stark contrast between the two countries. While 94% (CI: 92.9–95.1) of the population in India lives in a household in which no member has health insurance, only 8% (CI: 6.7–8.8) live in such households in China.

**Table 1:** Percentage distribution of population by the selected household characteristics, China and India, 2007–10.

		China (n = 9591)	India (n = 9583)
Household head education status	No formal schooling	14.8 (13.2,16.6)	31.9 (29.6,34.2)
	Primary	38.1 (35.7,40.6)	29.2 (27.5,31.0)
	Secondary	41.0 (39.3,42.6)	31.3 (29.5,33.3)
	College +	6.1 (4.3,8.6)	7.6 (6.5,8.9)
Wealth quintile	Poorest	20.0 (18.1,22.0)	20.0 (17.4,22.9)
	Poorer	20.0 (18.3,21.8)	20.0 (18.3,21.8)
	Middle	20.0 (18.9,21.2)	20.0 (18.6,21.5)
	Wealthier	20.1 (18.8,21.4)	20.0 (18.2,22.0)
	Wealthiest	19.9 (17.3,22.8)	20.0 (17.5,22.7)
Health insurance	No member	7.7 (6.7,8.8)	94.1 (92.9,95.1)
	Any member	92.3 (91.2,93.3)	5.9 (4.9,7.1)
Sex of the household head	Male	73.9 (71.7,75.9)	92.8 (91.9,93.7)
	Female	26.1 (24.1,28.3)	7.2 (6.3,8.1)
Age of the household head	< 50 years	21.2 (19.6,23.0)	45.1 (43.3,47.0)
	> = 50 years	78.8 (77.0,80.4)	54.9 (53.0,56.7)
Household member aged 50+	None	12.3 (11.5,13.2)	27.9 (26.4,29.4)
	1	27.3 (26.0,28.6)	34.8 (33.4,36.2)
	> = 2	60.4 (59.2,61.6)	37.4 (35.6,39.1)
Member aged ≤5 years	None	84.7 (83.2,86.2)	46.9 (44.7,49.0)
	> = 1	15.3 (13.8,16.8)	53.1 (51.0,55.3)
Household size	1	5.6 (4.9,6.5)	0.3 (0.2,0.4)
	2–3	56.2 (54.1,58.3)	6.6 (5.9,7.2)
	> = 4	38.2 (35.8,40.7)	93.1 (92.5,93.8)
Place of residence	Urban	49.9 (48.6,51.3)	26.1 (20.9,31.9)
	Rural	50.1 (48.7,51.4)	73.9 (68.1,79.1)
Inpatient care received	No	83.0 (81.8,84.1)	86.6 (85.3,87.8)
	yes	17.0 (15.9,18.2)	13.4 (12.2,14.7)
Outpatient care received	No	54.3 (51.7,56.9)	40.0 (37.6,42.5)
	yes	45.7 (43.1,48.3)	60.0 (57.5,62.4)
**Total**		**100.0**	**100.0**

*Note*: *95% CI given in parenthesis*.

The majority of the population in China and India lives in households headed by males: 74% (CI: 71.7–75.9) for China and 93% (CI: 91.9–93.7) for India. Also more than half of the population in these two countries lives in households whose heads are 50 years or older: 79% (CI: 77.0–80.4) for China and 55% (CI:53.0, 56.7) for India. In addition, most individuals live in households having at least one member who is 50 years or older. The average household is substantially larger in India, with more than 93% (CI: 92.5–93.8) of the population living in households of four or more.

In the case of inpatient care, about 17% (CI: 15.9–18.2) of the population in China and 13% (CI: 12.2–14.7) of the population in India belongs to households in which at least one member received in-patient care during the period covered in the survey. When it comes to outpatient care, about 60% (CI: 57.5–62.4) of the Indian population belongs to a household in which at least one member received outpatient care, which is substantially higher than the percentage in China (46%, CI: 43.1–48.3).

### Out-of-Pocket Health Expenditure

The mean share of out-of-pocket health expenditure (OOPHE) as a percentage of total as well as non-food expenditure (capacity to pay) is presented in Table 2. The share of OOPHE as a percentage of total expenditure is about 15% (CI: 14.4–15.8) in China and 12% (CI 11.0–12.1)in India. OOPHE as a percentage of capacity to pay is same for both China and India (23%, CI: 22.3–24.4 China; 22.3–24.1 India).

**Table 2:** Mean percentage share of out-of-pocket health expenditure (OOPHE) as a proportion of total expenditures and capacity to pay by selected household characteristics, China and India, 2007–2010.

		OOPHE’s share in total expenditure				OOPHE’s share in capacity to pay			
		China		India		China		India	
		% share (95% CI)		% share (95% CI)		% share (95% CI)		% share (95% CI)	
Household head Education status	No education	18.0 (16.4,19.6)	*	11.8 (11.0,12.7)	*	30.1 (27.9,32.2)	*	26.9 (25.5,28.4)	*
	Primary	16.3 (15.3,17.4)		10.9 (9.7,12.1)		25.1 (23.8,26.5)		22.4 (20.5,24.3)	
	Secondary	13.6 (12.8,14.5)		11.9 (11.0,12.8)		20.5 (19.3,21.6)		21.7 (20.3,23.1)	
	College +	10.7 (8.4,13.0)		11.2 (9.9,12.4)		15.7 (12.1,19.3)		17.4 (15.3,19.4)	
Wealth quintile	Poorest	16.9 (15.9,17.9)	*	11.1 (9.9,12.2)		30.4 (29.0,31.7)	*	28.3 (26.2,30.4)	*
	Poorer	17.5 (16.2,18.8)		11.1 (9.8,12.3)		27.5 (25.9,29.2)		25.0 (22.7,27.4)	
	Middle	15.3 (14.1,16.4)		11.3 (9.9,12.6)		22.2 (20.8,23.6)		23.1 (21.0,25.1)	
	Wealthier	15.3 (13.8,16.7)		12.2 (11.2,13.2)		21.4 (19.5,23.2)		21.6 (20.1,23.1)	
	Wealthiest	10.8 (9.5,12.0)		12.1 (11.0,13.3)		15.4 (13.7,17.1)		18.2 (16.6,19.8)	
Health insurance	No member	13.3 (11.8,14.9)		11.5 (10.9,12.0)		22.7 (20.6,24.9)		23.4 (22.5,24.4)	*
	Any member	15.3 (14.6,16.0)		12.5 (11.0,13.9)		23.4 (22.3,24.5)		20.2 (17.9,22.5)	
Sex of the household head	Male	15.1 (14.3,15.9)	*	11.6 (11.1,12.2)		23.2 (22.0,24.4)	*	23.3 (22.4,24.2)	
	Female	15.3 (14.4,16.2)		10. 4(9.0,11.8)		23.9 (22.5,25.2)		22.4 (19.5,25.4)	
Age of the household head	< 50 years	11.4 (9.9,12.9)	*	10.7 (10.1,11.4)	*	17.4 (15.4,19.5)	*	22.4 (21.1,23.6)	*
	> = 50 years	16.1 (15.5,16.8)		12.2 (11.4,13.0)		25.0 (24.0,25.9)		24.0 (22.8,25.2)	
Fifty plus aged elderly	No elderly	10.3 (9.3,11.3)	*	10.4 (9.7,11.2)	*	14.8 (13.5,16.2)	*	22.0 (20.6,23.5)	*
	One elderly	13.3 (12.2,14.4)		11.2 (10.4,12.0)		21.8 (20.3,23.2)		23.3 (21.9,24.7)	
	2+ Elderly	17.0 (16.0,17.9)		12.7 (11.6,13.7)		25.8 (24.5,27.1)		24.1 (22.8,25.4)	
Member aged ≤5 years	No child	15.4 (14.7,16.1)	*	10.6 (10.0,11.3)	*	23.6 (22.6,24.7)	*	20.6 (19.6,21.6)	*
	1+ child	13.6 (11.9,15.3)		12.3 (11.5,13.1)		21.9 (19.8,24.0)		25.6 (24.3,26.9)	
Household size	1	18.6 (16.8,20.4)	*	9.3 (5.9,12.7)		29.5 (26.9,32.1)	*	23.1 (17.1,29.1)	
	2–3	15.8 (15.0,16.5)		11.2 (10.0,12.5)		23.8 (22.8,24.7)		22.9 (20.8,25.0)	
	4+	13.7 (12.4,15.1)		11.6 (11.0,12.1)		21.9 (20.0,23.9)		23.3 (22.3,24.2)	
Place of residence	Urban	13.9 (12.9,15.0)		10.2 (9.3,11.2)	*	21.6 (20.0,23.3)	*	19.1 (17.6,20.6)	*
	Rural	16.3 (15.4,17.3)		12.0 (11.3,12.7)		25.1 (23.8,26.3)		24.7 (23.6,25.8)	
Inpatient care received	No	13.3 (12.6,14.1)	*	10.8 (10.3,11.4)	*	21.2 (20.1,22.3)	*	22.3 (21.4,23.2)	*
	Yes	23.9 (22.5,25.3)		16.1 (14.5,17.6)		33.9 (32.0,35.7)		29.2 (26.8,31.7)	
Outpatient care received	No	14.1 (13.2,14.9)	*	11.2 (10.4,12.1)		21.8 (20.7,22.8)	*	22.1 (20.7,23.5)	*
	Yes	16.4 (15.5,17.3)		11.7 (11.1,12.3)		25.3 (23.9,26.6)		24.0 (23.1,25.0)	
**Total**		**15.1 (14.4,15.8)**		**11.5 (11.0,12.1)**		**23.4 (22.3,24.4)**		**23.2 (22.3,24.1)**	

*Note*

* p≤0.*05*, *based on one way ANOVA*

*95% CI given in parenthesis*.

By educational status of household head OOPHE’s share decreases with the increase in educational status of household head for both China and India (except for the share as a percentage of total expenditure in India, where the trend is unclear). Moving to the pattern of OOPHE’s share with the wealth status, with the exception of OOPHE share as a percentage of total expenditure in India, OOPHE share shows a decreasing trend with increase in wealth. OOPHE’s share as a percentage of capacity to pay is lower among households in which at least one member has health insurance in India.

OOPHE as a percentage of total expenditure and as a percentage of capacity to pay is higher among those residing in female-headed households in China. It is also higher among those belonging to households with elder as the head and among households having at least an elderly member in both China and India. Interestingly, households with at least one child have a lower proportion of OOPHE in China but a higher proportion of OOPHE in India. There is a trend of an increasing proportion of OOPHE with decreasing household size in China.

Also, its share is higher in rural areas in India and in the case of share as a percentage of capacity to pay in China. OOPHE’s share in both China and India is higher among those from households’ where at least one member received inpatient and outpatient care compared to those from households’ where no member received them.

### Impoverishing Effects of OOPHE

The percentage of population falling below poverty line and the average deficit from the poverty line (poverty gap) due to OOPHE is presented in Table 3. About 7% (CI: 6.7–8.3) and 8% (CI: 7. 3–8.8) of the population falls below poverty line due to OOPHE in China and India, respectively.

**Table 3:** Percentage population falling poverty line (poverty head count ratio) and average deficit from the poverty line (poverty gap ratio) due to out-of-pocket health payments by selected household characteristics, China and India, 2007–2010.

		Poverty head count ratio (%)				Poverty gap ratio (%)			
		China		India		China		India	
		Poverty headcount (95% CI)		Poverty headcount (95% CI)		Poverty gap (95% CI)		Poverty gap (95% CI)	
Household head Education status	No education	9.6 (8.2,11.2)	*	9.7 (8.3,11.3)	*	2.9(2.2,3.6)	*	1.7(1.3,2.1)	*
	Primary	8.8 (7.8,10.1)		8.6 (7.0,10.5)		2.3(1.8,2.7)		1.4(1.0,1.9)	
	Secondary	6.1 (5.1,7.4)		6.9 (5.4,8.7)		1.5(1.2,1.8)		1.0(0.7,1.3)	
	College +	2.1 (1.1,4.3)		3.3 (1.9,5.8)		0.5(0.1,0.9)		0.4(0.1,0.6)	
Wealth quintile	Poorest	9.9 (8.1,12.0)	*	11.0 (9.3,13.0)	*	2.7 (2.1,3.2)	*	2.1 (1.5,2.6)	*
	Poorer	9.8 (8.3,11.6)		8.7(6.8,11.0)		2.8 (2.2,3.4)		1.4 (1.0,1.8)	
	Middle	7.5 (6.3,9.0)		9.1 (7.2,11.5)		2.1 (1.7,2.5)		1.5 (0.9,2.1)	
	Wealthier	6.1 (4.6,8.0)		8.0 (6.2,10.1)		1.4 (1.0,1.9)		1.0 (0.7,1.4)	
	Wealthiest	3.9 (2.9,5.2)		3.2 (2.2,4.8)		0.8 (0.6,1.0)		0.4 (0.2,0.6)	
Health insurance	No member	6.4 (5.2,7.8)		8.3 (7.6,9.2)	*	1.7(1.2,2.1)		1.3(1.1,1.5)	*
	Any member	7.5 (6.7,8.5)		2.7 (1.5,4.8)		2.0(1.7,2.2)		0.4(0.1,0.8)	
Sex of the household head	Male	7.6 (6.7,8.5)		8.1 (7.3,9.0)		2.0(1.7,2.3)		1.3(1.1,1.5)	
	Female	7.1 (5.7,8.8)		6.6 (4.7,9.3)		1.8(1.3,2.2)		1.0(0.5,1.5)	
Age of the household head	< 50 years	5.8 (4.5,7.5)	*	8.0 (6.8,9.4)		1.4(0.9,1.9)	*	1.3(1.0,1.6)	
	> = 50 years	7.9 (7.1,8.8)		8.0 (6.8,9.5)		2.1(1.8,2.4)		1.3(1.0,1.6)	
Fifty plus aged elderly	No elderly	5.0 (3.5,7.1)	*	8.3 (7.0,9.7)		1.2(0.7,1.6)	*	1.3(1.0,1.6)	
	One elderly	6.9 (5.9,8.0)		7.2 (6.1,8.5)		1.8(1.4,2.2)		1.2(0.9,1.5)	
	2+ Elderly	8.2 (7.2,9.3)		8.6 (7.1,10.4)		2.2(1.8,2.5)		1.4(1.0,1.7)	
Member aged ≤5 years	No child	7.2 (6.4,8.0)		6.9 (6.0,7.9)	*	2.0(1.7,2.2)		1.3(0.9,1.6)	*
	1+ child	8.9 (7.0,11.2)		9.0 (7.8,10.3)		2.0(1.2,2.7)		1.3(1.1,1.6)	
Household size	1	7.4 (6.1,9.0)		4.7 (2.3,9.4)		2.3(1.7,3.0)		1.1(0.3,1.9)	
	2–3	7.5 (6.6,8.4)		9.0 (6.9,11.7)		2.1(1.8,2.4)		1.6(0.9,2.3)	
	4+	7.4 (6.0,9.2)		7.9 (7.2,8.8)		1.8(1.3,2.2)		1.3(1.1,1.5)	
Place of residence	Urban	5.6 (4.4,7.0)	*	5.7 (4.3,7.6)	*	1.2(0.9,1.5)	*	1.0(0.6,1.5)	*
	Rural	9.3 (8.3,10.4)		8.8 (7.9,9.8)		2.7(2.3,3.1)		1.4(1.2,1.6)	
Inpatient care received	No	6.4 (5.7,7.3)	*	7.6 (6.8,8.5)	*	1.6(1.3,1.8)	*	1.2(1.0,1.4)	*
	Yes	12.3 (10.4,14.4)		10.5(8.5,13.0)		3.8(3.0,4.7)		1.9(1.3,2.5)	
Outpatient care received	No	7.1 (6.1,8.2)		7.7 (6.6,9.0)		1.8(1.5,2.1)		1.4(1.0,1.7)	
	Yes	7.9 (6.8,9.1)		8.2 (7.3,9.3)		2.1(1.8,2.5)		1.2(1.0,1.5)	
**Total**		**7.4 (6.7,8.3)**		**8.0 (7.3,8.8)**		**2.0(1.7,2.2)**		**1.3(1.1,1.5)**	

*Note*

* p≤0.*05*, *based on one way ANOVA*

*95% CI given in parenthesis*.

Regarding socioeconomic differentials in the percentage of population falling below poverty line due to OOPHE–percentage impoverished among the households with an uneducated household head is almost 10% (CI: 8.2–11.3) in both India and China which declines, respectively to 2.1% (CI: 1.1–4.3) in China and 3.3% (CI: 1.9–5.8) in India among the households headed by head who has at least a college level education. We would like to mention here that the present discussion should not be seen in any causal terms but only in terms of association. Also, about 9.9% (CI: 8.1–12.0) and 11% (CI: 9.3–13.0) of the poorest households, respectively in China and India fall below the poverty line due to OOPHE, compared to only 3.9% (CI: 2.9–5.2) and 3.2% (CI: 2.2–4.8) of the wealthiest households in China and India, respectively.

In India, relatively lower percentage (2.7%; CI: 1.5–4.8) of the households with insured members become impoverished compared to 8.3% (CI: 7.6–9.2) among the uninsured households in China. The proportion of households falling below poverty line increases with the increase in the number of fifty-plus aged elderly members in households in China. Whereas, in India, percentage impoverishment effect increases with the increase in under-5 aged children in the households. Proportion household falling below poverty line due OOPHE is higher in rural India and China compared with the urban area. In China, percentage impoverishment among the household (12.3%; CI: 10.4–14.4) with inpatient care is two times more of the household (6.4%; CI: 5.7–7.3) without inpatient care. Whereas, 10.5% (CI: 8.5–13.0) household receiving inpatient care fall below poverty line in India compared with 7.6% (CI: 6.8–8.5) among the household without inpatient care received member.

The average shortfall from the poverty line (poverty gap ratio) due to OOPHE for the Chinese and Indian population is also presented in Table 3. The average percentage shortfall in income for the population, from the poverty line due to OOPHE is 2% (CI: 1.7–2.2) in China and 1.3% (CI: 1.1–1.5) in India. There are substantial variations based on socioeconomic characteristics in the countries. Among the households with an uneducated household head, the poverty gap ratio is 2.9% (CI: 2.2–3.6) in China and 1.7% (CI: 1.3–2.1) in India, respectively, compared to less than half percentage among the households with a head having at least a college level education. Similarly, with the increase in household wealth status poverty gap ratio declines from 2.7% (CI: 2.1–3.2) in China and 2.1% (CI: 1.5–2.6) in India, respectively, among the poorest households to 0.8% (CI: 0.6–1.0) in China and 0.4% (CI: 0.2–0.6) in India, respectively, among the wealthiest households.

The poverty gap ratio is higher among those living in households headed by an elder (compared to a non-elderly) or having an elderly member in China. On the other hand the poverty gap ratio is higher among those living in households having at least one child in India.

In addition, the poverty gap ratio is higher among those living in rural areas in both China and India. The same is true for those living in households in which no member has health insurance in India. Similarly, the poverty gap ratio is higher among those belonging to households in which a member has received in-patient care in both China and India.

### Odds of Falling Below Poverty Line due to OOPHE

The adjusted socioeconomic differentials in odds of falling below poverty line due to OOPHE in China are presented in Table 4. Model-1 shows that households having a head who has completed secondary school and at least college level education is 29% (OR 0.71; CI: 0.54–0.94) and 73% (OR: 0.27; CI: 0.14–0.54), respectively less likely to be impoverished than the households headed by an uneducated head. By wealth quintile, the odds of falling below poverty line due to OOPHE decreases with increases in household wealth status. Households belonging to the wealthier and wealthiest quintile, respectively, are 38% (OR: 0.62; CI: 0.41–0.94) and 60% (OR: 0.40; CI: 0.27–0.59) less likely to be impoverished compared to the poorest households.

**Table 4:** Odds of becoming poor due to out-of-pocket health payments by selected household characteristics, China, 2007–2010.

		Model 1	Model2	Model3
Household head Education status	*No education*	*1*.*00*	*1*.*00*	*1*.*00*
	Primary	1.01(0.80,1.27)	1.02(0.81,1.29)	1.02(0.81,1.28)
	Secondary	0.71**(0.54,0.94)	0.84(0.64,1.12)	0.84(0.63,1.12)
	College +	0.27***(0.14,0.54)	0.39**(0.19,0.79)	0.39**(0.19,0.79)
Wealth quintile	*Poorest*	*1*.*00*	*1*.*00*	*1*.*00*
	Poor	1.00(0.78,1.28)	0.94(0.72,1.22)	1.02(0.74,1.4)
	Middle	0.77*(0.57,1.05)	0.72**(0.52,0.99)	0.81(0.53,1.23)
	Wealthier	0.62**(0.41,0.94)	0.56**(0.36,0.88)	0.56*(0.3,1.06)
	Wealthiest	0.40***(0.27,0.59)	0.35***(0.23,0.53)	0.48**(0.27,0.85)
Health insurance	*No member*	*1*.*00*	*1*.*00*	*1*.*00*
	Any member	1.41**(1.08,1.85)	1.13(0.81,1.59)	1.12(0.79,1.58)
Sex of the household head	*Male*		*1*.*00*	*1*.*00*
	Female		1.08(0.83,1.42)	1.08(0.83,1.42)
Age of the household head	*< 50 years*		*1*.*00*	*1*.*00*
	> = 50 years		1.01(0.77,1.33)	1.01(0.77,1.33)
Fifty plus aged elderly	*No elderly*		*1*.*00*	*1*.*00*
	One elderly		1.05(0.68,1.62)	1.06(0.68,1.63)
	2+ Elderly		1.29(0.79,2.1)	1.30(0.79,2.12)
Child age = <5 years	*No child*		*1*.*00*	*1*.*00*
	1+ child		1.30*(0.99,1.70)	1.30*(0.99,1.70)
Household size	*1 member*		*1*.*00*	*1*.*00*
	2–3 members		1.06(0.77,1.45)	1.05(0.76,1.44)
	4+ members		1.10(0.78,1.56)	1.09(0.77,1.54)
Place of residence	*Urban*		*1*.*00*	*1*.*00*
	Rural		1.56***(1.18,2.06)	1.56***(1.18,2.07)
Inpatient care received	*No*		*1*.*00*	*1*.*00*
	yes		2.11***(1.69,2.62)	2.56***(2.05,3.21)
Outpatient care received	*No*		*1*.*00*	*1*.*00*
	yes		1.23*(0.98,1.55)	1.33*(0.95,1.87)
Wealth quintile **×** Inpatient care received	Poorest *×* Yes			1.00
	Poor *×* Yes			0.85(0.54,1.36)
	Middle *×* Yes			0.71(0.37,1.35)
	Richer *×* Yes			0.91(0.44,1.90)
	Richest *×* Yes			0.53*(0.26,1.09)
Wealth quintile **×** outpatient care received	*Poorest × Yes*			*1*.*00*
	Poor *×* Yes			0.91(0.59,1.41)
	Middle *×* Yes			0.93(0.55,1.56)
	Richer *×* Yes			1.01(0.6,1.70)
	Richest *×* Yes			0.73(0.36,1.49)
**Constant**		0.09***(0.07,0.12)	0.05***(0.03,0.09)	0.05***(0.03,0.08)

*Note*

*p≤0.*10*

** p≤0.*05*

*** p≤0.*01*

*95% CI given in parenthesis*.

As we further adjust for the demographic characteristic and hospitalization in Model-2, the odds of the households having a head who has completed at least a college level education, compared to households with uneducated heads, becomes 0.39 (CI: 0.19–0.79). Households belonging to the middle, wealthier and wealthiest quintile are respectively 28% (OR: 0.72; CI: 0.52–0.99), 44% (OR: 0.56; CI: 0.36–0.88) and 65% (OR: 35; CI: 0.23–0.53) less likely to be impoverished than the poorest households. Ironically, in Model-1 households with any insured member were significantly more likely to be impoverished; however the difference becomes insignificant as we adjust for additional demographic factors (Model 2). Final Model-3 shows that socioeconomic differential in impoverishment effect of OOPHE remains same as in Model-2. Households having one or more under-5 aged children are 1.3 times (OR: 1.30; CI: 0.99–1.70) more likely to be impoverished compared to the households without any under-5 aged children. Further, households from the rural areas are 1.56 times more likely to be impoverished than the urban ones. Inpatient or outpatient care of the household members increases the odds of impoverishment with odds ratios being 2.56 (CI: 0.98–1.55) and 1.33 (CI: 0.95–1.87), respectively. Interaction result shows that, wealthiest households with inpatient hospitalization are 47% (OR: 0.53; CI: 0.26–1.09) less likely to impoverish compared to the poorest households with inpatient hospitalization.

Table 5 presents the adjusted socioeconomic differentials in impoverishment due to OOPHE for India. Model-1 is adjusted only for socioeconomic status, which shows that households headed by a member who has completed at least a college level education are 44% (OR: 0.56; CI: 0.29–1.07) less likely to fall below poverty line compared to the households headed by an uneducated member. Compared to the poorest households, wealthiest households are 66% (OR: 0.34; CI: 0.21–0.55) less likely to be impoverished due to OOPHE.

**Table 5:** Odds of becoming poor due to out-of-pocket health payments by selected household characteristics, India, 2007–2010.

		Model 1	Model2	Model3
Household head Education status	*No education*	*1*.*00*	*1*.*00*	*1*.*00*
	Primary	0.93(0.71,1.23)	0.97(0.72,1.31)	0.96(0.71,1.30)
	Secondary	0.84(0.59,1.20)	0.92(0.63,1.35)	0.92(0.63,1.35)
	College +	0.56*(0.29,1.07)	0.64(0.32,1.27)	0.63(0.32,1.25)
Wealth quintile	*Poorest*	*1*.*00*	*1*.*00*	*1*.*00*
	Poor	0.80(0.59,1.08)	0.78(0.58,1.06)	0.79(0.47,1.33)
	Middle	0.87(0.63,1.20)	0.81(0.59,1.13)	1.01(0.58,1.76)
	Wealthier	0.79(0.57,1.11)	0.73* (0.52,1.04)	1.10(0.65,1.89)
	Wealthiest	0.34*** (0.21,0.55)	0.30*** (0.18,0.48)	0.48* (0.21,1.11)
Health insurance	*No member*	*1*.*00*	*1*.*00*	*1*.*00*
	Any member	0.41*** (0.22,0.77)	0.42*** (0.22,0.77)	0.43*** (0.23,0.79)
Sex of the household head	*Male*		*1*.*00*	*1*.*00*
	Female		0.81(0.53,1.24)	0.82(0.54,1.26)
Age of the household head	*< 50 years*		*1*.*00*	*1*.*00*
	> = 50 years		0.98(0.61,1.58)	0.97(0.60,1.57)
Fifty plus aged elderly	*No elderly*		*1*.*00*	*1*.*00*
	One elderly		0.92(0.65,1.3)	0.93(0.65,1.31)
	2+ Elderly		1.12(0.7,1.77)	1.12(0.71,1.78)
Child age = <5 years	*No child*		*1*.*00*	*1*.*00*
	1+ child		1.28**(1.02,1.62)	1.30** (1.03,1.64)
Household size	*1 member*		*1*.*00*	*1*.*00*
	2–3 members		1.87(0.8,4.39)	1.89(0.8,4.48)
	4+ members		1.44(0.63,3.28)	1.47(0.64,3.37)
Place of residence	*Urban*		*1*.*00*	*1*.*00*
	Rural		1.44** (1.07,1.95)	1.44** (1.06,1.95)
Inpatient care received	*No*		*1*.*00*	*1*.*00*
	yes		1.54*** (1.18,2.01)	1.62* (0.93,2.81)
Outpatient care received	*No*		*1*.*00*	*1*.*00*
	yes		1.15(0.91,1.44)	1.52* (0.94,2.43)
Wealth quintile **×** Inpatient care received	*Poorest × Yes*			*1*.*00*
	Poor *×* Yes			1.26(0.52,3.02)
	Middle *×* Yes			1.20(0.54,2.64)
	Richer *×* Yes			0.63(0.27,1.48)
	Richest *×* Yes			0.55(0.16,1.92)
Wealth quintile **×** outpatient care received	*Poorest × Yes*			*1*.*00*
	Poor *×* Yes			0.92(0.49,1.73)
	Middle *×* Yes			0.64(0.32,1.29)
	Richer *×* Yes			0.57* (0.30,1.10)
	Richest *×* Yes			0.54(0.19,1.53)
**Constant**		0.13*** (0.1,0.16)	0.05*** (0.02,0.13)	0.04*** (0.02,0.12)

*Note*

*p≤0.*10*

** p≤0.*05*

*** p≤0.*01*

*95% CI given in parenthesis*.

Further, adjusting for demographic characteristics and inpatient & outpatient care of the household member in Model-2, the results show that there exists a significant economic differential in impoverishment due to OOPHE. Results show that, households belonging to the wealthier and wealthiest quintile are respectively 27% (OR: 0.73; CI: 0.52–1.04) and 70% (OR: 0.30; CI: 0.18–0.48) less likely to impoverish than the poorest households. Presence of any child younger than 5-year old increases the chances (OR: 1.28; CI: 1.02–1.62) of becoming impoverished compared to no presence of under-5 children. Inpatient hospitalization of any household member increases the odds (OR: 1.54; CI: 1.18–2.01) of impoverishments.

Findings in Model-3 show that the wealthiest households are 52% (OR: 0.48; CI: 0.21–1.11) less likely to fall below the poverty line than the poorest households. Also, health insurance of any household member reduces the chance of impoverishment by 0.57% (OR: 0.41; CI: 0.23–0.79). Rural households are 44% (OR: 1.44; CI: 1.07–1.95) more likely to fall below poverty line compared the urban households. Inpatient and outpatient care received by any household member increases the odds of impoverishment due to OOPHE, 1.62 (CI: 0.93–2.81) and 1.52 (CI: 0.94–2.43), respectively. Interaction results show that, wealthiest households with outpatient care received by any member are 43% (OR: 0.57; CI: 0.30–1.10) less likely to impoverish compared with the poorest household with outpatient care received.

The present study shows that given the demographic characteristics of the household and the health status of the household members; impoverishment effect of the OOPHE varies by the socio-economic status of the household. However, it may be argued that the present study does not take OOPHE as a linear predictor of impoverishment. There are primarily two reasons for not including OOPHE (or log OOPHE) as a predictor of impoverishment: *First*, there is one-to-one association between OOPHE and impoverishment, which means that impoverishment is directly related to health expenditure and as health expenditure (or OOPHE to be specific) will increase it will increase the chances of impoverishment and the objective of the present study is to find out the socio-economic differentials in the impoverishment effect of OOPHE. *Second*, even after ignoring one to one association between OOPHE and impoverishment, and introducing additional control using OOPHE quintile in the final Model-3 (results not shown but can be provided upon request) the socio-economic measures–wealth quintile and household head’s education remain significant.

## Discussion and Conclusions

We use data from the WHO’s Study on Global Ageing and Adult Health (SAGE) survey conducted during 2007–2010 to examine the socioeconomic differentials in the impoverishment effects of out-of-pocket health expenditure in China and India. We find that about 7% and 8% of the population falls below the poverty line due to OOPHE in China and India, respectively, with the proportion being significantly higher in the case of lower educational status of household’s head, lower wealth status of household, residence in rural areas and in-patient care in both China and India. In addition, the proportion is higher in the case of the household head being an elder or having an elderly member in the household in China. Furthermore, having one or more child in the household increase the chances of falling below poverty line due to OOPHE in India. Under-5 children from poor households are susceptible to infections and other diseases (due to their living conditions), therefore presence of under-5 children increases the chances of health care expenditure and therefore impoverishment in India [33–35].

Our estimate of the percentage of the population that falls below the poverty line due to OOPHE for China (7.4%) is similar to the estimate of Li et al. (2012) who reported that about 7.5% of the households in China fall below the poverty line due to OOPHE [16]. This difference is probably negligible and can be due to measurement error; however, it can also be due to the fact that the survey which we are using is nationally representative.

We also find that the average percentage shortfall in expenditure/income for the population from the poverty line (poverty gap ratio) due to OOPHE is 2% in China and 1.3% in India. Using National Sample Survey’s health expenditure survey 2004–05, Ghosh [8] has also shown a 1.8% poverty gap ratio for the Indian population. And, taking official poverty line Gustafsson and Li [36] estimated 3.9% poverty gap ratio in rural China. The socioeconomic differentials in poverty gap ratio are similar to the socioeconomic differentials in the proportion falling below the poverty line due to OOPHE.

Findings from the multivariate analysis indicate that lower wealth status and inpatient as well as outpatient care increase the odds of falling below poverty line significantly (with the extent much higher in the case of in-patient care) due to OOPHE in both China and India. Specifically, compared to outpatient care treatment, inpatient care received by any household member has much greater odds of pushing the household into poverty. In addition, having at least an under-5 child in the household, living in rural areas and having a household head with no formal education increases the odds of falling below poverty line significantly (compared to a head with college level education) due to OOPHE in China; whereas having at least a child, not having health insurance and residing in rural areas increases the odds of becoming poor significantly due to OOPHE in India.

Our findings have important policy implication for financing of healthcare in under-resourced country settings. Both China and India have seen unprecedented economic growth during the past two decades and are often considered the economic growth engine for the Global economy. However, as we have seen, both suffer from tremendous shortfalls when it comes to health care financing. In China, health insurance coverage has increased from 15% in 2000 to 96% in 2011 [16]. The Medical Insurance for Urban Employees (MIUE) scheme, designed exclusively for urban employees, is a mandatory programme based on cost sharing between employers and employees, with risk pooling managed at the municipal level whereas the Medical Insurance for Urban Residents scheme (MIUR) is for urban residents who are not covered by the MIUE and is co-financed by enrolees and local government [16,37,38]. The New Cooperative Medical Scheme (NCMS) is a voluntary programme based on cost sharing between government and farmers and covers mostly inpatient services and a few outpatient services [16,39]. However, despite China’s improvement in health insurance coverage, such coverage is not universal in terms of the definition of universal health coverage put forth by WHO [15,16,39,40]. The percentage of OOPHE in China increased from 20% in 1978 to more than 60% in 2001 [16,41]. The increased breadth of coverage, combined with low benefit levels, may have actually contributed to higher utilization rates and hence to a higher burden of out-of-pocket payments [16]. Though the number of people living in absolute poverty in China dropped to 27 million in 2011; impoverishment from medical expenses are still persistent. In 2004, 23.3% of rural households were impoverished by medical expenses [16,42]. Therefore as mentioned in Li et al. (2012) the policy-makers in China should focus on designing improved insurance plans by expanding the benefit package, redesigning cost sharing arrangements and provider payment methods and developing more effective expenditure control strategies [16].

Coming to India, about 400 million people in India live on less than $1.25 per capita per day and based on our analysis these poor people on average spend 11–15% of their total expenditure as OOPHE. As a share of total expenditure, OOPHE is high and policymakers should provide health insurance for the poor people in order to minimize their vulnerability to health and economic shocks. The other reason for such a high OOPHE is due to seeking care at private clinics [32,43,44]. More than three-quarters of health spending in India occurs at private facilities. Private facilities are chosen over public facilities because of better health infrastructure and quality of service at the private facilities. Policymakers should revamp the existing dysfunctional public hospitals which will help in shifting the demand from private to public hospitals and will require smaller OOPHE. More importantly, substantial investment is needed in the rural areas as more than 70% of the poverty-stricken population live in the rural areas and they are the one who are more susceptible to CHE in the absence of coping mechanisms. The implementation of the National Rural Health Mission and Rashtriya Swasth BimaYojana in India are welcome steps and if implemented efficiently may reduce the vulnerability faced by these poor people.

## References

[1] Bredenkamp C, Mendola M, Gragnolati M (2011) Catastrophic and impoverishing effects of health expenditure: new evidence from the Western Balkans. Health Policy and Planning 26: 349–356. 10.1093/heapol/czq070 20974750

[2] WHO (2014) Health financing: Health expenditure per capita Data by World Bank income group Global Health Observatory Data Repository. Geneva: World Health Organisation.

[3] Wagstaff A, van Doorslaer E (2003) Catastrophe and impoverishment in paying for health care: with applications to Vietnam 1993–1998. Health Econ 12: 921–934. 14601155 10.1002/hec.776

[4] Laveesh B, Peter B, Rajeev A (2010) The Impoverishing Effect of Healthcare Payments in India: New Methodology and Findings. Economic and Political Weekly XLV

[5] Kawabata K, Xu K, Carrin G (2002) Preventing impoverishment through protection against catastrophic health expenditure. Bull World Health Organ 80: 612 12219150 PMC2567587

[6] Alam K, Mahal A (2014) Economic impacts of health shocks on households in low and middle income countries: a review of the literature. Global Health 10: 21 10.1186/1744-8603-10-21 24708831 PMC4108100

[7] Pal R (2012) Measuring incidence of catastrophic out-of-pocket health expenditure: with application to India. Int J Health Care Finance Econ 12: 63–85. 10.1007/s10754-012-9103-4 22351126

[8] Ghosh S (2011) Catastrophic Payments and Impoverishment due to Out-of-Pocket Health Spending. Economic and Political Weekly XLVI.

[9] Leive A, Xu K (2008) Coping with out-of-pocket health payments: empirical evidence from 15 African countries. Bull World Health Organ 86: 849–856. 19030690 10.2471/BLT.07.049403 PMC2649544

[10] Kruk ME, Goldmann E, Galea S (2009) Borrowing and selling to pay for health care in low- and middle-income countries. Health Aff (Millwood) 28: 1056–1066. 19597204 10.1377/hlthaff.28.4.1056

[11] Flores G, Krishnakumar J, O'Donnell O, van Doorslaer E (2008) Coping with health-care costs: implications for the measurement of catastrophic expenditures and poverty. Health Economics 17: 1393–1412. 10.1002/hec.1338 18246595

[12] Balarajan Y, Selvaraj S, Subramanian SV (2011) Health care and equity in India. The Lancet 377: 505–515. 10.1016/S0140-6736(10)61894-6 PMC3093249 21227492

[13] Garg CC, Karan AK (2009) Reducing out-of-pocket expenditures to reduce poverty: a disaggregated analysis at rural-urban and state level in India. Health Policy and Planning 24: 116–128. 10.1093/heapol/czn046 19095685

[14] Li X, Shen JJ, Lu J, Wang Y, Sun M, Li C, et al (2013) Household catastrophic medical expenses in eastern China: determinants and policy implications. BMC Health Serv Res 13: 506 10.1186/1472-6963-13-506 24308317 PMC4234144

[15] Li Y, Wu Q, Liu C, Kang Z, Xie X, Yin H, et al (2014) Catastrophic Health Expenditure and Rural Household Impoverishment in China: What Role Does the New Cooperative Health Insurance Scheme Play? PLoS ONE 9: e93253 10.1371/journal.pone.0093253 24714605 PMC3979676

[16] Li Y, Wu Q, Xu L, Legge D, Hao Y, Gao L, et al (2012) Factors affecting catastrophic health expenditure and impoverishment from medical expenses in China: policy implications of universal health insurance. Bull World Health Organ 90: 664–671. 10.2471/BLT.12.102178 22984311 PMC3442391

[17] Van Doorslaer E, O'Donnell O, Rannan-Eliya RP, Somanathan A, Adhikari SR, Garg CC, et al (2007) Catastrophic payments for health care in Asia. Health Economics 16: 1159–1184. 17311356 10.1002/hec.1209

[18] Xu K, Evans DB, Kawabata K, Zeramdini R, Klavus J, Murray CJ (2003) Household catastrophic health expenditure: a multicountry analysis. Lancet 362: 111–117. 12867110 10.1016/S0140-6736(03)13861-5

[19] Van Doorslaer E, O'Donnell O, Rannan-Eliya RP, Somanathan A, Adhikari SR, Garg CC, et al (2006) Effect of payments for health care on poverty estimates in 11 countries in Asia: an analysis of household survey data. The Lancet 368: 1357–1364. 10.1016/S0140-6736(06)69560-3 17046468

[20] Kowal P, Chatterji S, Naidoo N, Biritwum R, Fan W, Lopez R, et al (2012) Data resource profile: the World Health Organization Study on global AGEing and adult health (SAGE). Int J Epidemiol 41: 1639–1649. 10.1093/ije/dys210 23283715 PMC3535754

[21] Xu K (2005) Distribution of health payments and catastrophic expenditures methodology Discussion Paper 2. Geneva: World Health Organization.

[22] Lu C, Chin B, Li G, Murray CJ (2009) Limitations of methods for measuring out-of-pocket and catastrophic private health expenditures. Bull World Health Organ 87: 238–244, 244a-244d. 19377721 10.2471/BLT.08.054379 PMC2654642

[23] Raban MZ, Dandona R, Dandona L (2013) Variations in catastrophic health expenditure estimates from household surveys in India. Bulletin of the World Health Organization 91: 726–735. 10.2471/BLT.12.113100 24115796 PMC3791647

[24] IIPS & WHO (2013) Study on global AGEing and adult health (SAGE) Wave 1: India National Report India, Mumbai: International Institute for Population Sciences.

[25] Haffner J, Moschel G, ten Horn GHMM (1987) Determination of the optimum Period of interview for retrospective collection of data. European archives of psychiatry and neurological sciences 236: 288–293. 3653150 10.1007/BF00380954

[26] Xu K (2003) Catastrophic health expenditure. The Lancet 362: 997. 10.1016/S0140-6736(03)14375-9 PMC4114756 14511936

[27] O'Donnell O, Doorslaer Ev, Wagstaff A, Lindelow M (2008) Catastrophic Payments for Health Care Analyzing Health Equity Using Household Survey Data. Washington, D.C: The World Bank.

[28] Van Minh H, Kim Phuong NT, Saksena P, James CD, Xu K (2013) Financial burden of household out-of pocket health expenditure in Viet Nam: Findings from the National Living Standard Survey 2002–2010. Social Science & Medicine 96: 258–263. 23246399 10.1016/j.socscimed.2012.11.028

[29] Boing AC, Bertoldi AD, Posenato LG, Peres KG (2014) The influence of health expenditures on household impoverishment in Brazil. Revista de Saúde Pública 48: 797–807. 25372171 10.1590/S0034-8910.2014048005113 PMC4211571

[30] Su TT, Kouyate B, Flessa S (2006) Catastrophic household expenditure for health care in a low-income society: a study from Nouna District, Burkina Faso. Bull World Health Organ 84: 21–27. 16501711 10.2471/blt.05.023739 PMC2626518

[31] Ruger JP, Kim HJ (2007) Out-of-pocket healthcare spending by the poor and chronically ill in the Republic of Korea. Am J Public Health 97: 804–811. 17395834 10.2105/AJPH.2005.080184 PMC1854861

[32] Buigut S, Ettarh R, Amendah D (2015) Catastrophic health expenditure and its determinants in Kenya slum communities. International Journal for Equity in Health 14: 46 10.1186/s12939-015-0168-9 25971679 PMC4438568

[33] Bhaskaram P (1987) Infections and malnutrition among poor children. The Indian Journal of Pediatrics 54: 535–545. 3308695 10.1007/BF02749048

[34] Kanjilal B, Mazumdar P, Mukherjee M, Rahman MH (2010) Nutritional status of children in India: household socio-economic condition as the contextual determinant. International Journal for Equity in Health 9: 19 10.1186/1475-9276-9-19 20701758 PMC2931515

[35] Pathak PK, Singh A (2011) Trends in malnutrition among children in India: Growing inequalities across different economic groups. Social Science & Medicine 73: 576–585. 21798638 10.1016/j.socscimed.2011.06.024

[36] Gustafsson B, Li S (2004) Expenditures on education and health care and poverty in rural China. China Economic Review 15: 292–301.

[37] Hu S, Tang S, Liu Y, Zhao Y, Escobar M-L, Ferranti D (2008) Reform of how health care is paid for in China: challenges and opportunities. The Lancet 372: 1846–1853. 10.1016/S0140-6736(08)61368-9 18930520

[38] Barber SL, Yao L (2010) Health insurance systems in China: A briefing note China: World Health Organization.

[39] Xu K, Saksena P, Fu XZH, Lei H, Chen N, Carrin G (2009) Health Care Financing in Rural China new Rural Cooperative Medical Scheme. Geneva: World Health Organisation (WHO).

[40] Meng Q, Xu L (2014) Monitoring and Evaluating Progress towards Universal Health Coverage in China. PLoS Medicine 11: e1001694 10.1371/journal.pmed.1001694 25243903 PMC4170954

[41] Zhang L, Liu N (2013) Health reform and out-of-pocket payments: lessons from China. Health Policy and Planning. 10.1093/heapol/czt006 23428367

[42] Cao S, Wang X, Wang G (2009) Lessons learned from China's fall into the poverty trap. Journal of Policy Modeling 31: 298–307.

[43] Nguyen H (2011) The principal-agent problems in health care: evidence from prescribing patterns of private providers in Vietnam. Health Policy and Planning 26: i53–i62. 10.1093/heapol/czr028 21729918

[44] Bonu S, Bhushan I, Rani M, Anderson I (2009) Incidence and correlates of ‘catastrophic’ maternal health care expenditure in India. Health Policy and Planning. 10.1093/heapol/czp032 19687135

