# Correction: Socio-Economic Differentials in Impoverishment Effects of Out-of-Pocket Health Expenditure in China and India: Evidence from WHO SAGE

**DOI:** 10.1371/journal.pone.0138499

**Published:** 2015-09-14

**Authors:** Kaushalendra Kumar, Ashish Singh, Santosh Kumar, Faujdar Ram, Abhishek Singh, Usha Ram, Joel Negin, Paul R. Kowal

There is an error in affiliation 3 for author Santosh Kumar. Affiliation 3 should be: Sam Houston State University, Huntsville, United States of America.
