# Correction: Correction: Socio-Economic Differentials in Impoverishment Effects of Out-of-Pocket Health Expenditure in China and India: Evidence from WHO SAGE

**DOI:** 10.1371/journal.pone.0148588

**Published:** 2016-01-29

**Authors:** The PLOS ONE Staff

## Notice of Republication

This correction was republished on January 19, 2016, to correct errors in the author byline. The publisher apologizes for the errors. Please download this correction again to view the correct version. The originally published, uncorrected correction and the republished, corrected correction are provided here for reference.

## Supporting Information

S1 File Originally published, uncorrected correction. (PDF)

S2 File Republished, corrected correction. (PDF)
