# Supplementary material for: Correction: Correction: Socio-Economic Differentials in Impoverishment Effects of Out-of-Pocket Health Expenditure in China and India: Evidence from WHO SAGE
Source: PLoS One. 2016 Jan 29;11(1):e0148588. doi: 10.1371/journal.pone.0148588 (PMC4732821; doi:10.1371/journal.pone.0148588)
Supplement: S1 File — (PDF) [file pone.0148588.s001.pdf]

PLOS | ONE

CORRECTION

Correction: Socio-Economic Differentials in Impoverishment Effects of Out-of-Pocket Health Expenditure in China and India: Evidence from WHO SAGE

Maria Romay-Barja, Inma Jarrin, Policarpo Ncogo, Gloria Nseng, Maria Jose Sagrado, Maria A. Santana-Morales, Pilar Aparicio, Basilio Valladares, Matilde Riloha, Agustin Benito

There is an error in affiliation 3 for author Santosh Kumar. Affiliation 3 should be: Sam Houston State University, Huntsville, United States of America.

Reference

1. Kumar K, Singh A, Kumar S, Ram F, Singh A, Ram U, et al. (2015) Socio-Economic Differentials in Impoverishment Effects of Out-of-Pocket Health Expenditure in China and India: Evidence from WHO SAGE. PLoS ONE 10(8): e0135051. doi:10.1371/journal.pone.0135051 PMID: 26270049

CrossMark
click for updates

OPEN ACCESS

Citation: Romay-Barja M, Jarrin I, Ncogo P, Nseng G, Sagrado MJ, Santana-Morales MA, et al. (2015) Correction: Socio-Economic Differentials in Impoverishment Effects of Out-of-Pocket Health Expenditure in China and India: Evidence from WHO SAGE. PLoS ONE 10(9): e0138499. doi:10.1371/journal.pone.0138499

Published: September 14, 2015

Copyright: © 2015 Romay-Barja et al. This is an open access article distributed under the terms of the Creative Commons Attribution License, which permits unrestricted use, distribution, and reproduction in any medium, provided the original author and source are credited.
